# Applications of Biosensors for Meat Quality Evaluations

**DOI:** 10.3390/s21227430

**Published:** 2021-11-09

**Authors:** Barbara Sionek, Wiesław Przybylski, Anita Bańska, Tomasz Florowski

**Affiliations:** 1 Institute of Human Nutrition Sciences, Warsaw University of Life Sciences (WULS), Nowoursynowska 159c str., 02-787 Warszawa, Poland; wieslaw_przybylski@sggw.edu.pl (W.P.); anita.banska@gmail.com (A.B.); 2 Institute of Food Sciences, Warsaw University of Life Sciences (WULS), Nowoursynowska 159c str., 02-787 Warszawa, Poland; tomasz_florowski@sggw.edu.pl

**Keywords:** pork, meat quality, biosensors, triglycerides, glucose, lactate

## Abstract

The aim of this study was to apply biosensors in the assessment of meat quality. The research was carried out on 20 samples of the *Longissimus* muscle obtained from pork of Polish Landrace and Polish Large White hybrids of fattening pigs. In the samples, 48 h after slaughter pH values, color parameters in the CIE system (L* a* b*), the volume of natural drip loss and intramuscular fat content were measured. The commercially available biosensor Accutrend Plus was used to measure glucose, triglycerides and lactic acid in meat juice. Significant (*p* ≤ 0.05) relationships between glucose, triglycerides, lactic acid levels and pork quality characteristics, i.e., pH (r = −0.62; r = −0.78; r = −0.68 respectively), natural drip loss and (r = 0.57; r = 0.58; r = 0.49), color parameters as L*, a* and b* (r = from 0.47 to 0.79) were demonstrated. The study showed a negative correlation between the intramuscular fat content and acidification of muscle tissue (r = −0.49), and a positive one with the brightness of color (r = 0.46). The results of the canonical analysis show that the measurement of all three metabolites in muscle juice allows the evaluation of the technological quality of meat with an accuracy of 86.54% (Rc = 0.93, *p* < 0.01).

## 1. Introduction

The intensification of livestock breeding and the emphasis on improving slaughter characteristics, such as an increase in meatiness and a decrease in the fat content of carcasses, have negative consequences, such as difficulties in maintaining meat quality and appropriate technological meat value. Genetic and environmental factors responsible for the occurrence of meat defects lead to economic losses in the meat industry. Due to numerous limitations, traditional methods of sensory evaluation, such as chemical and microbiological measurements, do not meet the requirements of a quick and objective assessment of meat quality [1]. Chemical methods, while objective and precise, are time consuming and require equipped laboratories and trained personnel. Similarly, microbiological counting methods require a very long time. Sensory analysis reveals some shortcomings, such as limited availability, high costs of an expert team and the inability to perform online measurements [2]. From this perspective, the unique properties of biosensors open up new possibilities for solving the problem of food quality and safety assessment. The most important features of biosensors are high sensitivity, short response time, the ability to work in real time, the capability to create integrated analytical systems, including the possibility of automatization and low production costs [3,4]. Biosensors are a promising and very useful tool for assessing the quality of meat and meat products. There are some reports of successful biosensor application in the evaluation of various characteristics of meat, the presence of microorganisms and other contaminants and the extent of glycolysis [5,6]. Non-invasive, fast, accurate and reliable tests to predict the quality of meat would facilitate the distribution of whole carcasses or cuts of carcasses according to their technological and quality characteristics. For this, a wide variety of biomarkers should be tested. There is a great need to identify predictive biomarkers or sets of parameters that are reliable indicators of meat quality. The strategy of the simultaneous assessment of meat characteristics (color, pH, intramuscular fat, volume of drip loss) and metabolites in muscle juice (glucose, lactate, triglycerides) can be a useful tool in the prediction of meat quality. An analysis of selected parameters and traits, and their correlation, could help to optimize meat production [7]. However, predicting the quality and functional properties of meats using selected parameters is a major challenge and should be confirmed in research. 

In a previous study, a commercially available biosensor was used to measure glucose and lactate content of meat juice. It was shown that the content of glucose and lactate was significantly related to the pH value, color parameters or natural drip loss [6]. Meat composition, e.g., fat content, is a very important quality trait that determines the sensory quality of meat. The amount of muscle fat is directly related to the content of triglycerides, which constitute 95% of meat lipids [8]. An increase in intramuscular fat content causes an increase in the volume of fat cells containing triglycerides, while the content of phospholipids, which are components of cell membranes, remains constant [9]. The firmness, tenderness and juiciness of meat depend on the amount of saturated fatty acids. The composition of fatty acids is associated with lipid oxidation reactions and is one of the most important parameters affecting the quality and technological usefulness of meat. The oxidative stability of meat depends on the balance of anti- and pro-oxidative compounds. Early identification of the quality class creates opportunities for proper raw meat management. For this reason, the meat sector needs to develop new, fast, easy-to-use methods for detecting meat defects. Since the 1970s, there has been a growing interest in the use of biosensors as measuring tools. Biosensors and the further development of their technology including the availability of nanomaterials could revolutionize existing analytical measurements. Due to their high selectivity, mobility, simple operation and lower costs compared to conventional laboratory methods, biosensors can be a valuable tool in assessing the quality of meat.

The aim of this study was to explore the possibility of using commercially available biosensors to assess the quality of pork. It has been hypothesized that the biosensors used to measure glucose, lactic acid and triglycerides could be effectively applied to measure these metabolites in natural meat drip loss, and that the results could be related to the technological quality of meat.

## 2. Materials and Methods

The research was carried out on the material of fattening pigs from a mass herd, which were hybrid Polish Landrace and Polish Large White. The slaughter of fattening pigs was carried out in the summer, in meat plants of the Mazovia Voivodeship. The animals were slaughtered following the European Union Council Regulations (EC) No 1099/2009 for the protection of animals at the slaughter. The body weight of fattening pigs was about 110 kg, and the meatiness was about 57%. The meat from fattening pigs was taken randomly from the longissimus dorsi muscle on the back behind the last rib towards the head. The research was carried out on 20 samples of pork. The obtained samples were transported to the laboratory in polystyrene containers at 4 °C. The samples (approx. 150 g each) were packed separately in vacuum bags.

### 2.1. Meat Color

The color of meat was measured 48 h post-mortem using a Konica Minolta CM-2300d (Konica Minolta, Inc., Tokyo, Japan), illuminant D65. Color parameters were determined in the CIE L*a*b* scale. Color measurements were performed in triplicate.

### 2.2. Natural Drip Loss

Drip loss was determined according to the method of Honikel (1998) by measuring the weight loss of the longissimus muscle (samples) as a percentage of the muscle weight [10]. At 48 h, post-mortem samples of *longissimus dorsi* muscle were placed in plastic bags and kept at 4 °C for 24 h. After that time, drip loss was collected into Eppendorf vials.

### 2.3. Muscle Glucose, Lactic Acid and Triglycerides Determination

Muscle glucose (mmol/L), lactic acid (mmol/L) and triglycerides (mg/dL) in drip loss were determined by the strip method using an Accutrend Plus apparatus (Roche Diagnostic GMBH, Mannheim, Germany). The tests were conducted using dedicated reactive strips. Samples were diluted with distilled water to obtain the appropriate analyte concentration. The test results were obtained at: 60 s (lactic acid); 12 s (glucose); and 174 s (triglycerides) after placing a 20 µl drop on a corresponding reactive strip. Each test was performed in two repetitions.

### 2.4. Glycolytic Potential

Muscle glycolytic potential (GP) was calculated according to the formula proposed by Monin and Sellier (1985). The sum of glucose and lactic acid is calculated, and expressed as mmol lactic acid per liter of fresh muscle exudate [11].

### 2.5. pH

The pH value was measured 48 h after slaughter (pH_48_) using a pH meter from WTW, model 340i (Wilheim, Germany) with temperature compensation and with a SenTix® SP electrode, number 103645 (Wilheim, Germany). The pH meter was calibrated before measurements using standard phosphate buffers (pH 4 and pH 7). Measurements were made directly in muscle tissue in two repetitions, at different points of the tissue.

### 2.6. The Intramuscular Fat Content

The determination of the fat content was carried out by the Soxhlet method in accordance with the PN-ISO 1444:2000 Standard for the determination of intramuscular fat content for meat and meat products [12]. The principle of the method is to extract the dried sample with n-hexane or light petroleum and then remove the residual solvent by evaporation, and then to dry and weigh the extracted fat.

### 2.7. Determining the Meat Quality Classes

Meat quality classes, such as RFN (red, firm and nonexudative), DFD (dark, firm and dry) and RSE (red, soft and exudative) were determined on the basis of the pH values, drip loss and lightness L* values according to Koćwin-Podsiadła et al. (2006) [13].

### 2.8. Statistical Analysis

The collected results, obtained during all measurements, were subjected to statistical analysis in Statistica version 13.0 (TIBCO Software Inc. (2017). Statistica (data analysis software system), version 13. http://statistica.io (accessed on 10 May 2021)). For average values and standard deviation, minimum and maximum values were calculated. Between the studied traits, Pearson’s simple correlation coefficients were calculated. The significance of the calculated coefficients is given for the *p* < 0.05 level. Canonical analysis was used to assess the links between the set of traits characterizing the quality of meat and the set of parameters measured by biosensors. It enabled the assessment of the relationship between two groups of variables [14]. As a set of variables explained, the characteristics of meat quality were designated and measurements made with biosensors were used as a set of explanatory variables. 

## 3. Results and Discussion

### 3.1. Meat Quality Classes

Based on obtained test results, the identification of meat quality classes was determined in the pork samples according to Koćwin-Podsiadła et al. (2006) [13]. Normal pork quality (RFN: red, firm, nonexudative) was found in 25% of the samples, while 75% of the samples showed the characteristics of defective meat. Four samples of meat (20%) of dark red color (lowest value L* = 44.98), low natural drip loss (smallest size 0.4%) and higher pH (maximum pH = 6.03) were classified as a defect DFD (dark, firm, dry). The brightness parameter (L*) of another four samples of meat (20%) was within the limits for normal raw material (*52–58). The volume of drip loss and post-mortem acidification of these samples were as expected for pale and exudative meat and they were identified as RSE (red, soft, exudative) [13]. In 35% of pork samples, the quality defect was a pale pink color with high values of spectrophotometric measurement results (maximum L* = 63.80). The high incidence of pale meat may have been associated with the higher environmental temperature during the summer season in which the study was conducted. In a publication by Gajana et al. (2013), a higher incidence of pale pink meat classified as PSE was also found during the summer; this defect occurred in 43% of the pig carcasses tested during this period, while DFD accounted for 7% [15]. Occurrence of the above-mentioned pork quality classes in the tested material reflects a wide range of muscle juice drip loss, where the minimum loss was 0.4% and the largest was 7% of the mass. Differences between the samples in the amount of natural drip loss obtained 48 h after slaughter were also observed by Sieczkowska et al. (2017) in their study of pork obtained from the mass population [16].

The range of variability of features such as pH and color parameters, as well as glycolytic potential, confirms the presence of defective meat (Table 1). The meat quality characteristics were similar to the results obtained by Kamiński et al. (2010), Traore et al. (2012) and Żelechowska et al. (2012) [17,18,19].

### 3.2. Applicability of Biosensors for Measurements of Biochemical Parameters in Meat Drip Loss

The selection of research material is of significant importance for both the proper assessment and the usefulness of biosensors; in the present work, it was muscle leakage. In a study conducted by Čobanović et al. (2020), blood obtained from pigs at slaughter was used for biochemical analysis for the assessment of pork quality [7]. It was shown that the concentration of glucose in the slaughter blood was poorly positively correlated with the lean meat content. The increased level of lactate and glucose had a weak effect on the acidification and color of the meat. According to the authors, weak and inconsistent correlations between meat quality and lactate and glucose content probably result from the use of whole blood for analysis, which resulted in an underestimation of their concentration in meat. Di Luca et al. (2013) stated that muscle exudate provides valuable information about the pathways and processes underlying the post-mortem ageing period [20]. Hargreaves et al. (2009) presented a method that involved quantifying glycogen in muscles based on acid hydrolysis. Glucose measurements were performed with the strip test and a home glucometer [21]. The results indicate that the use of a glucometer to determine glucose in the drip loss of muscle tissue is a convenient, fast and reliable method. In the publication by Przybylski et al. (2016), the relations between glucose and lactic acid, determined by biosensors in meat juice, and the glycolytic potential in the longissimus dorsi muscle, assessed by enzymatic hydrolysis, were evaluated [22]. A fairly high correlation between these sets of traits was shown, r = 0.87 (*p* < 0.001). In the present study, we expanded the experiment by adding the assessment of triglycerides in drip loss. The results of the biosensor strip test for the measurement of glucose, triglycerides and lactic acid in the meat juice in studied meat samples are presented in Table 1. 

### 3.3. Correlation of Drip Loss Biochemical Parameters with Meat Quality

Table 2 shows Pearson’s simple correlation coefficients between the tested meat quality characteristics and the metabolites measured by biosensors in natural drip loss. The results of the study showed the existence of significant relationships between the level of glucose, lactic acid and pH value, and color parameters and natural drip loss. Similar results were obtained by Przybylski et al. (2016) and Buła et al. (2019) [22,23]. The correlation coefficient values presented in Table 2 indicate that the measurement of glucose and lactic acid in drip loss may be a good indicator of quality when related to muscle tissue acidification, color parameters or natural drip loss. 

The Pearson’s correlation showed a positive correlation between glucose concentration, triglyceride levels, lactic acid levels, natural drip loss and L* parameters of meat color. A higher glucose level was related to a lower ultimate pH, lighter as well as more red and yellow meat and higher natural drip loss. Similar results were obtained by Choe et al. (2008) and Choe and Kim (2014) [24,25]. These authors also showed that higher glucose content was accompanied by higher lactic acid content. The highest and significant correlations of the glycolytic potential with all measured variables (except intramuscular fat) confirm that glycolytic potential is a very good indicator of meat quality [13,26]. In the case of pH values, the higher degree of acidification in the tissue, the lower the values reached by the above variables (Table 2). A negative correlation between lactic acid and pH levels (−0.78) was also described in a study by Sieczkowska et al. (2013), in which the measurement of lactic acid from the longissimus lumborum muscle was described as one of the most practical determinants in the diagnosis of acidic and exudative meat (PSE, ASE) [26]. In the present study, similar average results (Table 1) were obtained for the level of lactic acid, pH and the L* component of the color, such as in the study by Sieczkowska et al. (2013) [26]. The analysis of the impact of variables on pork meat quality described by Przybylski et al. (2015) showed a negative correlation between glucose levels in blood serum and pH values [27]. This relationship was observed in our own results (−0.62). There was also a positive correlation between glucose and color brightness (0.69), and natural drip loss (0.57) and glycolytic potential. Moreover, it has been shown that higher triglyceride concentrations are associated with lower carcass meatiness.

No significant relationship was observed between the measurement of triglyceride content in the natural drip loss and the content of intramuscular fat. However, the level of triglycerides in the drip loss was significantly correlated with the pH value, natural drip loss and glucose and lactic acid content (Table 2). So far, no research has been conducted on the measurement of triglycerides in a natural drip loss and therefore it is difficult to compare results with the literature data. Nevertheless, Przybylski et al. (2015) showed a significant positive relationship between the thickness of subcutaneous fat and the content of triglycerides in the blood serum of pigs. In addition, fatteners with higher blood glucose levels also had higher triglycerides levels [27]. On the other hand, Yu et al. (2007) and Przybylski et al. (2009) observed lower triglyceride levels in pigs with low intramuscular fat content in the longissimus dorsi muscle and vice versa, i.e., pigs with higher triglyceride levels had a higher intramuscular fat level [28,29]. The relationship between blood plasma and muscle levels has been also confirmed by Mortimer and Przybylski (2016) [30]. In the conducted study, a negative correlation was observed between the content of intramuscular fat and the pH value (−0.49). An increase in fat content was accompanied by an increase of L* color parameter (0.46) (Table 2). The reverse relationship was obtained in studies conducted by Czarniecka-Skubina et al. (2007), where a higher content of intramuscular fat was seen in meat with a higher ultimate pH and darker color [31]. These different data may be the result of testing genetically different materials.

In the studied material, the correlation between muscle acidification and intramuscular fat and triglycerides was negative (−0.49 and −0.68) and it can be concluded that both are related. A negative correlation was observed between fat content and pH value (−0.49), while with the increase in fat content, the L* parameter of color (0.46) also increased (Table 2). The reverse relationship was obtained in a study conducted by Czarniecka-Skubina et al. (2007), where a higher content of intramuscular fat and a higher ultimate pH was accompanied by the darker color of meat [31]. 

### 3.4. Lipids Impact on Meat Quality 

Lipids ensure the appropriate tenderness, juiciness and taste of meat products [32]. Fat content and composition are very important to consumers. However, the degradation and oxidation of lipids leads to deterioration of the quality of meat and meat products. In the tested pork samples, the parameter that showed the greatest variation was the concentration of triglycerides; its content ranged from 715 mg/dL to 2865 mg/dL (Table 1). In the study by Przybylski et al. (2015), in raw meat obtained from crosses of Naïma gilts with boars P76 of the P76-PenArLan hybrid line, no such large deviations were observed between individual samples, and the weight of the fattening pig was positively correlated with the thickness of the lard and the lipid profile, i.e., the concentration of triglycerides and HDL cholesterol [27]. In the present study, no significant correlations were observed between the fat content of the tissue and the concentration of triglycerides determined in muscle juice (Table 2). In a study by Čobanović et al. (2020), the level of lactate in the blood obtained at the slaughter of the pigs was negatively correlated with the fat content and showed a weak positive correlation with lumbar muscle thickness and lean meat content [7]. 

The differences in the results obtained by different researchers reflect the complexity of meat quality traits, which are influenced by many factors, including: genetic background; breeding; environmental factors; nutrition; ante mortem conditions; and slaughter procedures [33]. The growth and fattening of animals are associated with an increase in fat deposition, first in subcutaneous fats and later in muscle tissue. Muscle fat (intramuscular) cannot be removed before consumption, and therefore has an impact on the quality of the product and human health. The differences in muscle fat content can be significant between pigs [34]. In addition, the amount of triglyceride in the muscles is strongly related to the total fat content and ranges from 0.2% to over 5% [35]. The intramuscular fat content depends on the different metabolic and cellular pathways involved in adipogenesis. The observed variability in the amount of intramuscular fat depends on the expression of genes regulating the development of adipocytes during animal growth. This hypothesis may explain the problems with finding biomarkers with satisfactory predictive ability for meat quality and showing correlations with the content of intramuscular fat [36]. 

### 3.5. Multivariate Relationship 

The canonical analysis was used to estimate the relationship between the measurement of all three biochemical parameters—glucose, lactate and triglycerides (GLT)—with all tested characteristics of the technological quality of meat. Measurements of GLT in drip loss were adopted as a set of explanatory variables, while the traits characterizing the quality of meat were adopted as a set of explained variables (Table 3). The results of the calculations showed that the canonical correlation coefficient reached the value of Rc = 0.93 (*p* < 0.01) and the determination coefficient reached R^2^_C_ = 86,54% (Table 3). These results indicate that all parameters taken together have a higher diagnostic value than individually. Similar conclusions about the prognostic value of glycolytic enzymes determined in natural drip loss were reported by Przybylski et al. (2016) and Sierra et al. (2012) [22,37]. The studies of these authors confirmed that glycolytic changes occurring in muscle tissue after slaughter play a decisive role in shaping the quality of pork, and the indicators related to them have a great diagnostic value in the assessment of meat quality. Koćwin-Podsiadła et al. (2006) showed that canonical correlation between traits characterized as glycolytic potential (glycogen, glucose, lactate), measured in vivo or post-mortem, and meat quality characteristics such as pH, ATP degradation, meat color, water holding capacity and technological yield of meat during curing and cooking were Rc = 0,81 and Rc = 0,95 respectively [13]. This is in line with the results presented in Table 3.

The data in Table 3 regarding the canonical variables indicate that the greatest impact on the obtained results was the association of pH, color parameters and drip loss with GLT, and second was the association of the level of triglycerides with pH and the color parameter a* and b*. Additionally, there is also the association of triglycerides with a color parameter of b*. The analysis of canonical weights confirms the above observations; they indicate that the greatest contribution to the creation of canonical variables was made by such variables as pH, color parameters, glucose, lactic acid and triglycerides (Table 4). These results confirm that the set of muscle juice metabolic parameters—glucose, lactate and triglyceride content—increase the diagnostic yield of meat quality assessment.

The application of reliable indicators to predict meat quality is one of the main challenges for the meat industry. A very important issue is the selection of animals capable of producing meat with good sensory and technological quality. There is a need to develop new indicators useful in improving breeding and slaughter practices [36]. Based on a multi-parameter biosensor assessment, it seems possible to create an automated, integrated meat classification system that would significantly reduce costs and improve the accuracy of meat quality classification [38]. 

## 4. Conclusions

The use of biosensors in predicting meat quality can be comparably effective to traditional analytical methods. Analyses with biosensors are simplified and time saving; furthermore, the amount of research material needed is small and does not force a violation of the product structure. 

A positive correlation was shown between triglyceride levels, glucose, lactic acid and the degree of natural drip loss and the L*, a* and b* color components, indicating the usefulness of a multi-parameter biosensor assessment in determining meat quality.

More experiments should be performed to evaluate the biochemical parameters of muscle juice in different pork quality and processed meat in different environments. 

The use of biosensor technology can significantly improve meat quality assessment and reduce the cost of testing in meat plants and slaughterhouses. However, further work is required to develop new indicator standards characterizing quality classes and defects.

## Figures and Tables

**Table 1:** Characteristics of studied materials.

Traits	Mean	S.D.	Min.	Max.
pH	5.52	0.17	5.29	6.03
Color parameters:				
L*	55.52	5.54	44.98	63.80
a*	3.42	1.68	1.01	6.48
b*	13.89	1.85	10.28	16.96
Drip loss—DL (%)	3.10	1.61	0.43	7.00
Intramuscular fat—IMF (%)	3.30	1.33	1.16	6.04
Glucose—G (mmol/L)	8.63	2.84	3.00	13.89
Lactate—La (mmol/L)	105.65	12.24	65.00	121.00
Glycolytic potential (mmol/L)	122.90	16.58	71.00	141.78
Triglycerides—Tg (mg/dL)	1888.50	584.98	715.00	2865.00

**Table 2:** Pearson’s simple correlation coefficients between the examined traits.

Traits	L*	a*	b*	DL	IMF	G	La	GP	Tg
pH	−0.52 *	−0.01	−0.40	−0.63 *	−0.49 *	−0.62 *	−0.78 *	−0.79 *	−0.68 *
L*	−	0.22	0.77 *	0.51 *	0.46 *	0.69 *	0.50 *	0.60 *	0.23
a*		−	0.57 *	0.14	−0.15	0.47 *	0.40	0.45 *	−0.03
b*			−	0.45 *	0.30	0.79 *	0.53 *	0.66 *	0.20
DL				−	0.29	0.57 *	0.58 *	0.63 *	0.49 *
IMF					−	0.35	0.35	0.38	0.25
G						−	0.67 *	0.84 *	0.47 *
La							−	0.97 *	0.65 *
GP								−	0.64 *
Tg								−	−

Abbreviations: color parameters—L*, a*, b*; DL—drip loss; IMF—intramuscular fat content; G—glucose; La—lactate; GP—glycolytic potential; Tg—triglycerides. * Coefficient significant at *p* < 0.05.

**Table 3:** The results of the canonical analysis: factor structure.

Traits	Variables Explained		
	V1	V2	V3
pH	−0.80	−0.53	−0.14
L*	0.71	−0.35	0.30
a*	0.54	−0.48	−0.52
b*	0.80	−0.51	0.28
Drip loss—DL (%)	0.67	0.23	0.32
Intramuscular fat—IMF (%)	0.41	0.05	0.09
	**Explanatory Variables**		
	**U1**	**U2**	**U3**
Glucose—G (mg/dL)	0.91	−0.22	0.34
Lactate—La (mmol/L)	0.90	0.35	−0.24
Triglycerides—Tg (mg/dL)	0.54	0.74	0.41
Canonical correlation coefficient R_C_	0.93 **		
Coefficient of determination R^2^_C_	86.54%		

** Coefficient significant at *p* < 0.01.

**Table 4:** The results of the canonical analysis: canonical weights.

Traits	Variables Explained		
	V1	V2	V3
pH	−0.58	−0.92	0.23
L*	0.07	−0.49	−0.34
a*	0.38	−0.17	−1.30
b*	0.23	−0.44	1.33
Drip loss—DL (%)	0.08	0.15	0.32
Intramuscular fat—IMF (%)	0.06	−0.11	−0.32
	**Explanatory Variables**		
	**U1**	**U2**	**U3**
Glucose—G (mg/dL)	0.57	−0.88	0.84
Lactate—La (mmol/L)	0,60	0.33	−1.40
Triglicerydes—Tg (mg/dL)	−0,12	0.93	0.91

## Data Availability

Not applicable.
